# Schizophrenia is a disorder of aberrant neurodevelopment: A synthesis of evidence from clinical and structural, functional and neurochemical brain imaging studies

**DOI:** 10.4103/0019-5545.37663

**Published:** 2007

**Authors:** Ganesan Venkatasubramanian

**Affiliations:** Department of Psychiatry, National Institute of Mental Health and Neurosciences, Bangalore - 560 029, Karnataka, India

## SCHIZOPHRENIA AS A DISORDER OF ABERRANT NEURODEVELOPMENT

Several reasons have been advanced to support the view that schizophrenia is a neurodevelopmental disorder.[1] The primary reason is that the onset of schizophrenia has a cumulative age incidence distribution or developmental function, that is nonlinear with a peak change in slope or acceleration that usually takes to occur during young adulthood. Given the plausibility of the existence of brain abnormalities in schizophrenia at the onset of the illness, it further seems reasonable to conceive the onset of schizophrenia as a neurodevelopmental disorder. Further support has been provided by epidemiological studies showing premorbid intellectual deficits dating back to the early development[2] and neuropathological studies showing altered cerebral cytoarchitecture indicative of a developmental rather that acquired encephalopathy.[3]

The main neurodevelopmental hypotheses for schizophrenia set forth in the last 10 years are relatively restricted and share three assumptions:[4]

1. The primary pathogenetic defect is an early derangement of the orderly development of the central nervous system that occurs in the pre- or perinatal period;
2. The period of active operation of the causative agent is of short duration, meaning that it is essentially static;
3. The behavioral consequences of this static process remain relatively latent until long after the primary pathogenetic process has run its course.

Furthermore, aspects of illness suggesting neurodevelopmental deviation may not apply to all individuals and some authors have argued for neurodevelopmental and non-neurodevelopmental subtypes of the disorder[5] and even neurodegenerative subtypes of the disorder.[6]

However, neurodegenerative hypotheses of schizophrenia have been criticized mainly because of the following reason: gliosis, which is regarded as a necessary neuropathological hallmark of neuronal degeneration, has not been found in a number of carefully controlled postmortem studies in patients with schizophrenia. These neuropathological studies provide strong evidence against a classic neurodegenerative pathogenesis of schizophrenia.[1,4] In addition, the failure of the first prospective CT studies of schizophrenia to show the same age-disproportionate progressive increase in ventricle-brain ratio after onset of illness that is seen in disorders such as Huntington's disease also rejects neurodegeneration.[4]

Some researchers have proposed another alternative that schizophrenia is a progressive neurodevelopmental disorder. This postulates that developmental mechanisms, such as apoptosis and pruning, can continue to go awry over many years without resulting in excessive gliosis. This accounts for both the neuropathological findings implicating prenatal pathology and the imaging findings (especially the recent Magnetic Resonance Imaging studies that report evidence for progressive brain changes in schizophrenia).[4] Thus, although schizophrenia is argued by a few as a neurodegenerative disorder,[6,7] majority of the existing evidence points towards a neurodevelopmental etiopathogenesis.[1,4]

## NEURODEVELOPMENTAL HYPOTHESIS OF SCHIZOPHRENIA - EVIDENCE BASE

The tenets of the neurodevelopmental hypothesis of schizophrenia derive from observations of epidemiological, clinical and brain imaging evidence for neurodevelopmental deviance.[8] These include studies on schizophrenia patients examining obstetrical complications, age-at-onset (AAO), sex differences, minor physical anomalies (MPAs), neurological soft signs (NSS) and structural brain abnormalities. Other stigmata include abnormal dermatoglyphics and childhood neuromotor precursors of adult schizophrenic illness. This research paper attempts at providing an overview of evidence for neurodevelopmental basis for schizophrenia with specific focus on highlighting relevant Indian studies supporting this concept.

### Neurodevelopmental Schizophrenia - Minor Physical Anomalies

Minor physical anomalies are defined as minor structural deviations, which are found in numerous areas of the body (head, eyes, ears, mouth, hands and feet) and which are of little general physiological or cosmetic importance.[9] MPAs are considered to represent “indelible fingerprints” of fetal maldevelopment during early pregnancy. Firstly, MPAs develop during the first and possibly early second trimesters of pregnancy, a period of major development of the brain. Secondly, MPAs are typically found in the ectoderm and thus share their embryonic origin with that of the brain.[10] Thus, “*the underlying brain and the overlying face are inexorably intertwined*” in the course of early development.[11] An increase in the frequency of MPAs in individuals with a given form of psychopathology would, thus, represent prima facie evidence for early developmental origins of the psychopathology. Studies have shown that increased rates of MPAs are found in a broad range of mental, behavioral and physical disorders with neurodevelopmental abnormality.[12]

At least 12 different studies have found increased rates of MPAs in adults with schizophrenia,[10] whereas one study failed to find a significant increase in patients.[13] Earlier Indian studies have found significantly increased MPAs in patients with schizophrenia than normal population.[14,15] Recent Indian study examining MPAs has shown significantly increased prevalence of MPAs in antipsychotic-naïve, first-episode schizophrenia patients in comparison to age, sex, education and socio-economic status matched healthy control subjects.[16] The evidence of excessive MPAs points towards aberrant neurodevelopmental antecedents in schizophrenia.[12]

### Neurodevelopmental Schizophrenia - Age-At-Onset

Neurodevelopmental disorders tend to have more severe manifestations in males compared to females, i.e., the AAO being earlier in males with comparatively worse outcome.[17] The AAO of schizophrenia in males is earlier by 3-5 years compared with females. This is a robust finding across many studies.[18] Recent Indian study suggested that the differential AAO might be a function of perinatal brain insult supporting deviant neurodevelopmental antecedents in schizophrenia.[19]

In addition, the definition of a developmental phenomenon has been reserved to those characteristics that show a nonlinear mean developmental function across age in some group that is relatively consistent across birth cohorts.[17] In the ABC Study by Hafner *et al.*,[20,21] based on the first sign of a mental disorder, males showed a single marked peak of onset between ages 15 and 25 followed by a steady decline and females showed a lower and broader peak between ages 15 and 30 and with a second smaller peak between ages 45 and 49. Such a nonlinear relationship of the onset across age is another strong pointer towards neurodevelopmental basis. Therefore, it is well established that schizophrenia have poorer outcome in males when compared to females.[17,18,22]

### The relationship between age-at-onset and minor physical anomalies

In a study by Green *et al.*,[23] schizophrenia group with onset at or before 18 years had significantly more subjects with MPAs. An Indian study has examined the relationship between AAO and MPAs by comparing schizophrenia patients with younger AAO and older AAO. It was observed that schizophrenia patients with younger AAO had significantly more MPAs than those with older AAO.[16] These findings suggest that early-onset schizophrenia is associated with a more compromised central nervous system probably due to neurodevelopmental insult.

### Neurodevelopmental schizophrenia - Neurological soft signs

Clinical neurological abnormalities in schizophrenia have been described since the time of Kraepelin.[24] These abnormalities, which include persistence of primitive reflexes, abnormalities in motor coordination, motor sequencing and sensory integration, are traditionally called as “neurological soft signs” (NSS). NSS are considered as manifestations of developmental insult to the brain. The prevalence of NSS in patients with schizophrenia has been estimated on review by Heinrichs and Buchanan[25] to be between 50% and 65%, compared with 5% in control groups. Earlier Indian study on medicated schizophrenia patients has reported similar prevalence of NSS.[15] Most of the previous studies have examined NSS in schizophrenia patients on chronic neuroleptic treatment.[26] NSS have been reported to occur secondary to antipsychotic exposure.[27] Examination of first-episode, antipsychotic-naïve schizophrenia patients can avoid these confounding factors. Recent Indian study on first-episode, antipsychotic-naïve Indian schizophrenia patients has shown significantly increased prevalence of NSS in comparison to matched healthy controls.[28]

### Neurodevelopmental schizophrenia - Cerebellar soft signs (CSS)

Recent findings provide new evidence suggesting the so-called “soft” neurological signs might have some measure of neuroanatomical validity.[29] Cerebellar neurological abnormalities have been recently reported to be associated with poorer premorbid functioning, severe negative symptoms, impaired cognitive performance and smaller cerebellar volume. In fact, it has been suggested that cerebellar dysfunction leading to cognitive dysmetria may provide a unifying explanation for the diverse motor, cognitive and symptomatic manifestations of schizophrenia.[30,31]

Indian study examining CSS in antipsychotic-naïve schizophrenia patients has shown interesting results. CSS scores were significantly higher in patients in comparison with healthy controls. Discriminant analysis revealed two CSS subscores to be significant accounting for 78% of classification. CSS total score, posture subscore and oculomotor subscore had significant positive correlation with a negative syndrome score.[32]

## NEUROLOGICAL AND CEREBELLAR SOFT SIGNS SUPPORT NEURODEVELOPMENTAL ETIOPATHOGENESIS

NSS may be an intrinsic component of schizophrenia because of their presence even in first-episode patients without antipsychotic treatment.[26,28,33] In addition, there was no significant correlation between NSS scores and illness duration in antipsychotic-naïve schizophrenia patients; this suggests a nonprogressive probably neurodevelopmental pathogenesis.

## NEUROIMAGING STUDIES IN SCHIZOPHRENIA

The modern era of neurodevelopmental research in schizophrenia began in 1976, when Johnstone *et al.*,[34] showed that some chronic schizophrenics had enlarged cerebral ventricles on computerized tomography (CT). Following CT studies estimated the prevalence of neurodevelopmental focal lesions [aqueduct stenosis, arachnoid and septal cysts and agenesis of the corpus callosum (CC)] between 6% and 9% in schizophrenia.[35]

In the late 1980s, the first wave of MRI studies essentially aimed at replicating the findings already established with CT. A second wave of MRI studies reported a measurable reduction in gray matter volume in medial temporal lobe structures including hippocampus-amygdala and parahippocampal gyrus. Another meta-analysis of 40 volumetric MRI studies concluded that regions, such as the amygdala and hippocampus, were probably smaller. In addition, volumes of the frontal lobe, parahippocampus, thalamus and superior temporal gyri are decreased in patients when compared with controls.[36] Recent studies examining cortical gray matter volume suggest there is a 5%-8% global reduction in cortical gray matter volume in schizophrenia. Several of the studies have failed to find a correlation between length of illness and degree of volumetric reduction. This suggests again that this change is developmental rather than degenerative.[35]

Current thinking about the mechanisms of schizophrenia postulates a disruption in distributed functional circuits rather than a single abnormality in a single brain region such as prefrontal cortex.[35] Schizophrenia-related size differences in the cerebrum have been particularly associated with the heteromodal association areas, especially the prefrontal, temporal and inferior parietal cortex.[37]

No specific group of regions has yet emerged as the “schizophrenia circuit”, but a consensus is developing on some of the nodes that may be involved. These nodes include various subregions within the frontal cortex (orbital, dorsolateral, medial), the anterior cingulate gyrus, the thalamus, several temporal lobe subregions,[38] basal ganglia[39] and cerebellum.[40] It appears that brain structures with established anatomical connectivity with heteromodal association cortices and having delayed maturation (thus being at high to suffer neurodevelopmental insult) are likely to be abnormal in schizophrenia. Interestingly, studies on CC in schizophrenia offer support to this possibility.

Corpus callosum is the major interhemispheric connecting tract with definitely connections with multiple heteromodal association areas. Clinical and experimental data suggest that the CC is part of the highest order-latest maturing neural network of the brain.[41] Reports of an association between psychosis and dysgenesis of the CC have suggested that neurodevelopmental damage to this structure might have been causally related to the psychosis.[42] Review of the association between developmental defects of the CC and psychosis suggests probable neurodevelopmental etiopathogenesis.[43] Studies examining CC in schizophrenia including a meta-analysis of 11 studies[44] have shown that patients have a decreased global size of CC. Studies on regional CC abnormalities clarify that the size reduction preferentially involves fibers connecting the heteromodal association cortices.[44]

### Neuroimaging studies in schizophrenia: Indian scenario

Most of the prior neuroimaging studies have examined chronic schizophrenia patients on long-term neuroleptic treatment. In addition, they have used thicker MRI slices, which might have resulted in potential partial volume effects. In an attempt to avoid these potential confounds and to examine the status of brain structure in Indian schizophrenia patients, MRI and MRS studies using 1 mm slices (compared to 3 and 5 mm slices used in most of the western studies) were performed on antipsychotic-naïve, first contact schizophrenia patients in comparison with age, sex, education, handedness matched healthy volunteers were performed at the National Institute of Mental Health and Neurosciences, Bangalore. These studies have examined the following brain structures using semi-automated morphometric techniques in antipsychotic-naïve schizophrenia patients in comparison with matched healthy subjects:

1. Corpus callosum[16,45–47]
2. Cerebellar vermis[48]
3. Caudate nucleus[49,50]

Very recently, another Indian study has utilized a fully automated, unbiased brain image analysis technique called Voxel Based Morphometry (VBM) to examine antipsychotic-naïve Indian schizophrenia patients.[51] Further, the neurochemical correlates of brain structural abnormalities have been examined using Magnetic Resonance Spectroscopy.[52,53]

## STRUCTURAL BRAIN IMAGING STUDIES

The areas of CC and cerebellar vermis were found to be smaller in patients when compared with controls.[47] This is in support of the findings in a recent meta-analysis of CC size in schizophrenia patients.[44] The reduction in the size of CC was restricted mainly to anterior CC and body of CC.[47] This finding supports a previous study by Woodruff *et al*.[54] The CC and cerebellar vermis abnormalities were found only in younger AAO but not in older AAO patients.[16,45] In addition, the caudate nucleus volume was found to be reduced in schizophrenia patients.[49,50] A recent automated image analysis study has confirmed these volumetric brain structural abnormalities. In addition to this, it has also demonstrated volume reduction in heteromodal association cortices, limbic cortices and thalamus in schizophrenia patients.[51]

### Neurochemical brain imaging studies of basal ganglia in schizophrenia

Earlier efforts at detecting metabolic changes in the striatum with ^31^P MRS were limited and produced conflicting findings, perhaps due to differences in the MRS methodology and medication status of the patients studied among other reasons. A ^31^P MRS study examining basal ganglia metabolism in antipsychotic-naïve Indian schizophrenia patients showed statistically significant decreased phosphocreatine/total phosphorous and phosphocreatine/total ATP ratios in both basal ganglia of the patient group as compared to the matched normal subjects suggesting increased metabolism in the basal ganglia.[53] These metabolic abnormalities were associated with smaller caudate volume and younger onset of psychosis.[55] Another study examining membrane phospholipid abnormalities has shown antipsychotic-naïve schizophrenia patients to have significantly and bilaterally elevated levels of phosphomonoester/phosphodiester ratios in basal ganglia as compared to controls.[52] A recent study that has examined the relationship between MRS abnormalities and developmental reflexes in schizophrenia patients has shown that schizophrenia patients with developmental reflexes had the lowest phosphocreatine/total ATP ratio in basal ganglia indicating more severe metabolic abnormality. In addition, patients with developmental reflexes had younger AAO of psychosis.[56]

## STRUCTURAL AND NEUROCHEMICAL ABNORMALITIES SUPPORT NEURODEVELOPMENTAL HYPOTHESIS

None of these brain structural abnormalities had significant correlation with illness duration pointing towards nonprogressive probably developmental pathogenesis. The observation of these brain structural abnormalities at the time of illness onset prior to the antipsychotic treatment and lack of correlation with illness duration supports neurodevelopmental pathogenesis in schizophrenia.

### From phenomenology to brain pathology: Neurophenomenology, neurocognitive models and neurodevelopmental schizophrenia

Neurophenomenology proposes that the phenomenological accounts of the structure of experience and their counterparts in cognitive science relate to each other through reciprocal constraints.[57]

Such a phenomenological approach that is informed by neuroscience would help in synthesizing neurocognitive models of schizophrenia. Perhaps the most widely cited and influential of these is the neurocognitive model proposed by Christopher Frith.[58] This model hypothesizes positive symptoms of schizophrenia to result from dysfunction of self-monitoring system and negative symptoms to result from defective spontaneous willed action. For example, first-rank symptoms like somatic passivity might be secondary to parietal lobe abnormalities, whereas spontaneous willed action deficits might result from hypofrontality.[58] Recent functional MRI (fMRI) study has reported Schneiderian first-rank symptoms to be associated with parietal lobe hyperactivity in schizophrenia patients.[59] Another fMRI study has reported temporal randomness (which is an indicator of spontaneous willed action) to correlate with frontal lobe dysfunction in schizophrenia patients.[60] Interestingly, a recent pharmacological fMRI study has shown some of these brain dysfunctions appear to improve with Modafinil, a cognitive enhancer.[61]

Interestingly, both these brain areas (frontal and parietal lobes) are among those that have been reported to be at high risk for being affected by neurodevelopmental insult. Frontal lobe one of the important neo-cortical area is also among the latest to mature and hence is at high risk for neurodevelopmental insult.[1] The function of parietal association cortices is improved by estrogen[62] and estrogen has been reported to have a neuroprotective effect during developmental stages.[63] Postpartum period, which is associated with major alterations in estrogen levels, is associated with a high risk for developing schizophrenia. Indeed, postpartum onset schizophrenia has been described in the literature.[64,65] Put together, these observations suggest parietal lobe to be at high risk for neurodevelopmental insult.

In addition to positive and negative symptoms, obsessive-compulsive symptoms in schizophrenia might also indicate neurodevelopmental insult. Schizophrenia patients with obsessive-compulsive symptoms have been reported to have significantly earlier AAO that those without obsessive-compulsive symptoms.[66] Obsessive-compulsive symptoms in schizophrenia have been reported to be associated with first-rank symptoms.[66,67] Schizophrenia patients with obsessive-compulsive symptoms have significantly more brain abnormalities and more NSS pointing towards the neurodevelopmental etiopathogenesis.[68] These observations of clinical and brain structural correlates of phenomenology suggest probable neurodevelopmental etiopathogenesis in schizophrenia.

## NEURODEVELOPMENTAL PATHOGENESIS OF SCHIZOPHRENIA: CONCLUSIONS AND FUTURE DIRECTIONS

In conclusion, selective review of relevant studies shows schizophrenia patients to have more MPAs, NSS, CSS, structural and neurochemical brain abnormalities in comparison with matched healthy controls. Neurophenomenological correlates of schizophrenia point towards abnormalities involving brain regions that are at high risk for neurodevelopmental insult. Observation of these abnormalities in multiple domains of aberrant neurodevelopmental indicators with lack of correlation with illness duration strongly suggests neurodevelopmental etiopathogenesis in schizophrenia.

Future studies are required in the following areas to establish and explore the clinical utility of neurodevelopmental model of schizophrenia:

1. Longitudinal studies to elucidate the exact trajectorial profile of these brain changes;
2. Studies on high risk subjects to examine for the predictive validity of clinical (e.g., NSS and MPAs) and brain structural indicators of aberrant neurodevelopment with relevance to the future occurrence of psychosis;
3. Pharmacological imaging studies to explore the potential and the neural basis for the beneficial effects of novel molecules (cognitive enhancers and nutritional supplements, for instance, polyunsaturated fatty acids especially in the context of membrane phospholipid abnormalities in schizophrenia) in improving the course and possibly to prevent the onset of schizophrenia.

While these “ontogeny” related studies might help in understanding the “proximal” mechanisms of schizophrenia pathogenesis, it is important that the “distal” mechanisms need to be understood. Thus, one needs to apply the principles of “evolutionary psychiatry” to enable comprehensive understanding of the “Human Ontogenic and Phylogenic Evolutionary” basis of schizophrenia.
